# Supplementary material for: Hormonal and Reproductive Factors in Relation to Cardiovascular Events in Women with Early Rheumatoid Arthritis
Source: J Clin Med. 2022 Dec 27;12(1):208. doi: 10.3390/jcm12010208 (PMC9820904; doi:10.3390/jcm12010208)
Supplement: Supplementary file 1 [file jcm-12-00208-s001.zip › jcm-2069971-supplementary.pdf]

Supplementary Table S1. Included diagnoses defined as cardiovascular event (CVE).

Cerebrovascular events	
I63.0–6, I63.8-9, 164.9, 165.0–3, 165.8–9	Cerebral ischemic infarction
G45.3	Amaurosis fugax
G45.9	Transient cerebral ischemic attack
Acute coronary syndrome	
I21.0–4, I21.9, I22.0–1, I22.8–9, I24.8-9	Acute myocardial infarction, subssequent ST elevation (STEMI) and non-ST elevation (NSTEMI) myocardial infarction
I20.0	Unstable angina pectoris
Angina with presence of intervention	
I20.1, I20.8–9 + Z95.1 or Z95.5 or FNG02 or FNC96 or FNG05 or FNC-10, -20, -30, -40, -50, -60	Angina pectoris together with presence of aortocoronary bypass graft or coronary angioploasty implant graft or PCI, PTCA, stent
